# Stereotactic Percutaneous Electrochemotherapy as Primary Approach for Unresectable Large HCC at the Hepatic Hilum

**DOI:** 10.1007/s00270-021-02841-1

**Published:** 2021-05-25

**Authors:** L. Luerken, M. Doppler, S. M. Brunner, H. J. Schlitt, W. Uller

**Affiliations:** 1 Department of Radiology, University Hospital Regensburg, Franz-Josef-Strauss-Allee 11, 93053 Regensburg, Germany; 2 Department of Surgery, University Hospital Regensburg, Franz-Josef-Strauss-Allee 11, 93053 Regensburg, Germany; 3 Faculty of Medicine, Department of Diagnostic and Interventional Radiology, Medical Center, University of Freiburg, Hugstetter Straße 55, 79106 Freiburg, Germany

**Keywords:** Percutaneous Electrochemotherapy, Interventional Oncology, Percutaneous Ablation, Non-thermal Ablation, Stereotactic Navigation, HCC

## Abstract

Electrochemotherapy (ECT) is a novel non-thermal ablative technique that combines chemotherapy and the application of electric pulses for reversible cell membrane electroporation. This method was recently performed in the treatment of deep-seated liver tumors during open surgery but experience about percutaneous ECT is rare and further developments like combination of percutaneous ECT with stereotactic navigated devices may be very promising. We report on a case of a 4.7 × 4.5 × 3.5 cm unresectable HCC at the hepatic hilum adjacent to the major vessels and the bile duct that was successfully treated using percutaneous ECT in combination with stereotactic navigation. Follow-up imaging 6 weeks and 6 months after ECT showed complete response.

## Introduction

In recent years, local ablative procedures have become a curative treatment option even for patients with increasingly large hepatocellular carcinoma (HCC). This is due to technical advances in (1) thermal ablative procedures such as multiantenna microwave ablation (MWA) [1, 2], (2) combination of radiofrequency ablation (RFA) or MWA with transarterial chemoembolization (TACE) [3] and (3) the use of stereotactic navigation devices [4–6]. Irreversible electroporation (IRE) is a non-thermal treatment method, which allows treatment of HCC lesions in close proximity to major blood vessels without the disadvantages of thermal ablation methods like heat sink effect and without the risk of injury of central vessels and bile ducts [7]. This renders IRE a good treatment option for tumor lesions close to the liver hilum. However, this method is limited to tumors with a maximum diameter of about 3 cm.

Resulting from the abovementioned limitations percutaneous ablation can be very challenging, especially in case of a large central tumors.

Electrochemotherapy (ECT) is a non-thermal ablative technique that combines chemotherapy and the application of electric pulses for reversible cell membrane electroporation. Consequently, mitotic tumor cell death results through high intracellular concentrations of the chemotherapeutic agent. In contrast to ECT, IRE uses irreversible cell membrane electroporation (without the need of additional drugs) that results in damage to the cell membrane and apoptosis. ECT has proven to be a safe and effective treatment for cutaneous tumors/metastases and first case series state, that ECT is also an effective treatment method for liver lesions in intraoperative settings [8–10].

We report a case of ECT for treatment of a large central HCC using a stereotactically navigated percutaneous approach.

## Patient and Technique

**Fig. 1:**
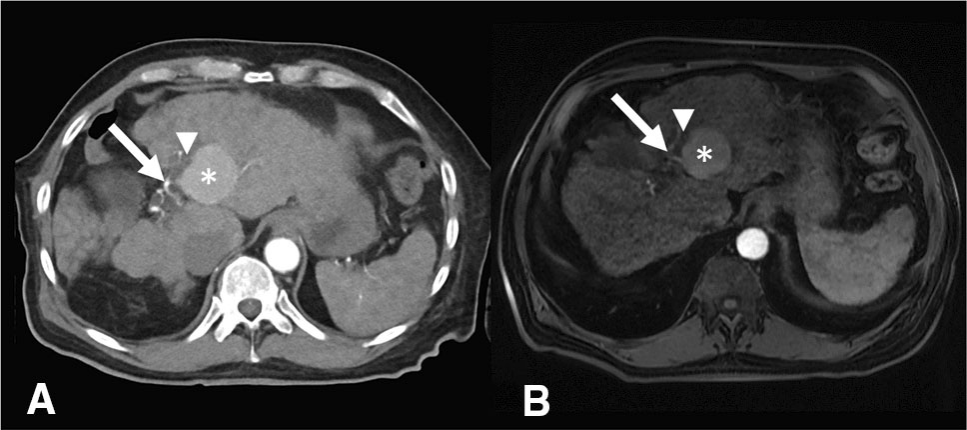
75-years old male patient with Child A liver cirrhosis and a 4.7 × 4.5 × 3.5 cm large central HCC BCLC B involving liver segments IV/VIII (asterisks). **A** Contrast-enhanced CT in arterial phase. **B** Contrast-enhanced MRI, T1 vibe 3d fs in arterial phase. Arrows mark close proximity of the tumor to the liver artery; Arrowheads mark close proximity of the tumor to the central bile ducts, resulting in local cholestasis

A 75-years old male patient with Child A liver cirrhosis presented with a 4.7 × 4.5 × 3.5 cm large central HCC involving liver segments IV/VIII and a satellite HCC (1.3 × 1.3 × 1.2 cm, segment V). The portal vein showed thrombosis resulting in cavernous transformation while the hepatic artery and the central bile ducts were located in close proximity to the HCC resulting in local cholestasis (Fig. 1). Alpha-fetoprotein showed a slight elevation with 9.3 ng/ml. The case was reviewed in our multidisciplinary tumor board (consisting of hepatologists, oncologists, liver transplant surgeons, interventional radiologists, radiation therapists, nuclear medicine physicians and pathologists). The decision to perform percutaneous ECT of the central lesion was made because of the large size and the proximity to the hepatic artery and central bile duct. MWA of the satellite tumor was scheduled because of its size of less than 3 cm and a more peripheral location.

### Technique of Stereotactic Percutaneous Electrochemotherapy

**Fig. 2:**
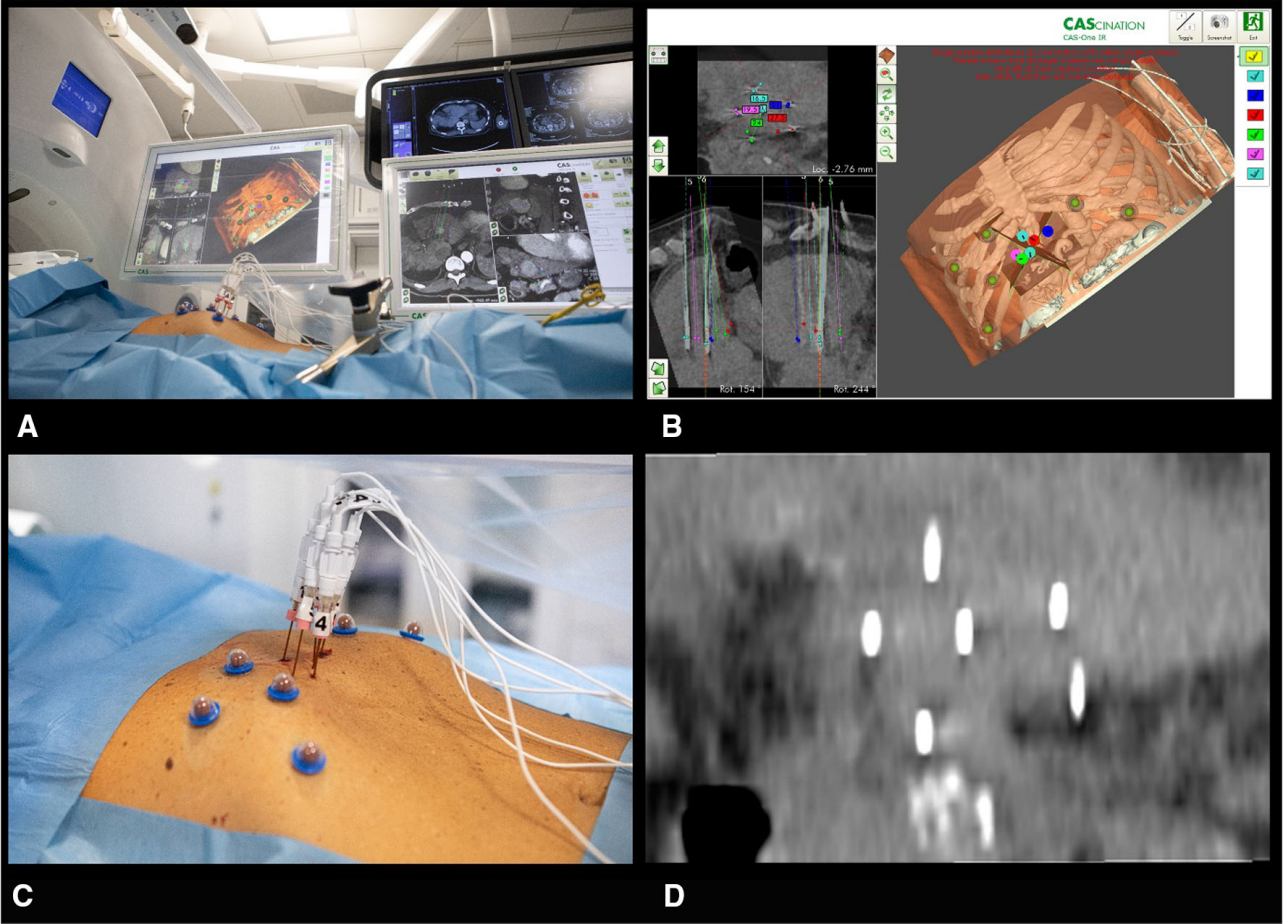
Technical details of the procedure. **A** Treatment setting in the interventional CT suite using stereotactic navigation (Cascination CAS-ONE IR). **B** Planning and validation of electrode placement, using the 3D model, generated by the navigation software. **C** Percutaneous placement of 6 ECT-electrodes (IGEA Cliniporator VITAE). **D** MPR of the electrode tips in a native control scan demonstrates spacing of the electrode-pairings between 2.1 and 3 cm

The procedure was performed under general anesthesia and deep muscle relaxation in the supine position with the right side slightly lifted. A Somatom Definition Edge CT-scanner (Siemens, Forchheim, Germany) was used in combination with a stereotactic navigation system (CAS-ONE IR, CAScination AG, Bern, Switzerland) for optimized percutaneous placement of ECT electrodes (IGEA Cliniporator VITAE, IGEA S.p.A., Carpi, Italy). Induction of general anesthesia, patient preparation and setup of the stereotactic navigation system took 37 min. Six electrodes with 4.0 cm active tip were placed within and around the tumor with the distance between the electrode-tips ranging from 2.1 to 3.0 cm (Fig. 2). Stereotactic placement of the 6 ECT-electrodes took 59 min, including planning of the trajectories. Bleomycin was selected as chemotherapeutic agent because, unlike other drugs for ECT, it can be administered intravenously [11]. 30000 IU of Bleomycin diluted in 10 ml NaCl (15000 IU/m^2^ body surface area) was injected intravenously (as bolus); 8 min after Bleomycin injection, 8 electric pulses with a frequency of 1000 Hz, impulse length of 100 µs, and voltages ranging from 1.4 to 3 kV were delivered between each electrode pairing over a timespan of 16 min. Stereotactic MWA of the satellite tumor was performed during the same session. A control-scan in arterial and portal-venous contrast phase showed complete devascularization of both, tumor and satellite. No periinterventional adverse events occurred. Total procedural time (including patient preparation, time under anesthesia, ECT and MWA of the satellite tumor) took 3 h and 24 min.

### Early Postinterventional Course

Magnetic resonance imaging (MRI) performed 24 h after the procedure confirmed complete devascularization of tumor and satellite. The patient was discharged two days after the procedure in good condition and without complications. Alpha-fetoprotein decreased to 7.55 ng/ml.

### Follow-up

**Fig. 3:**
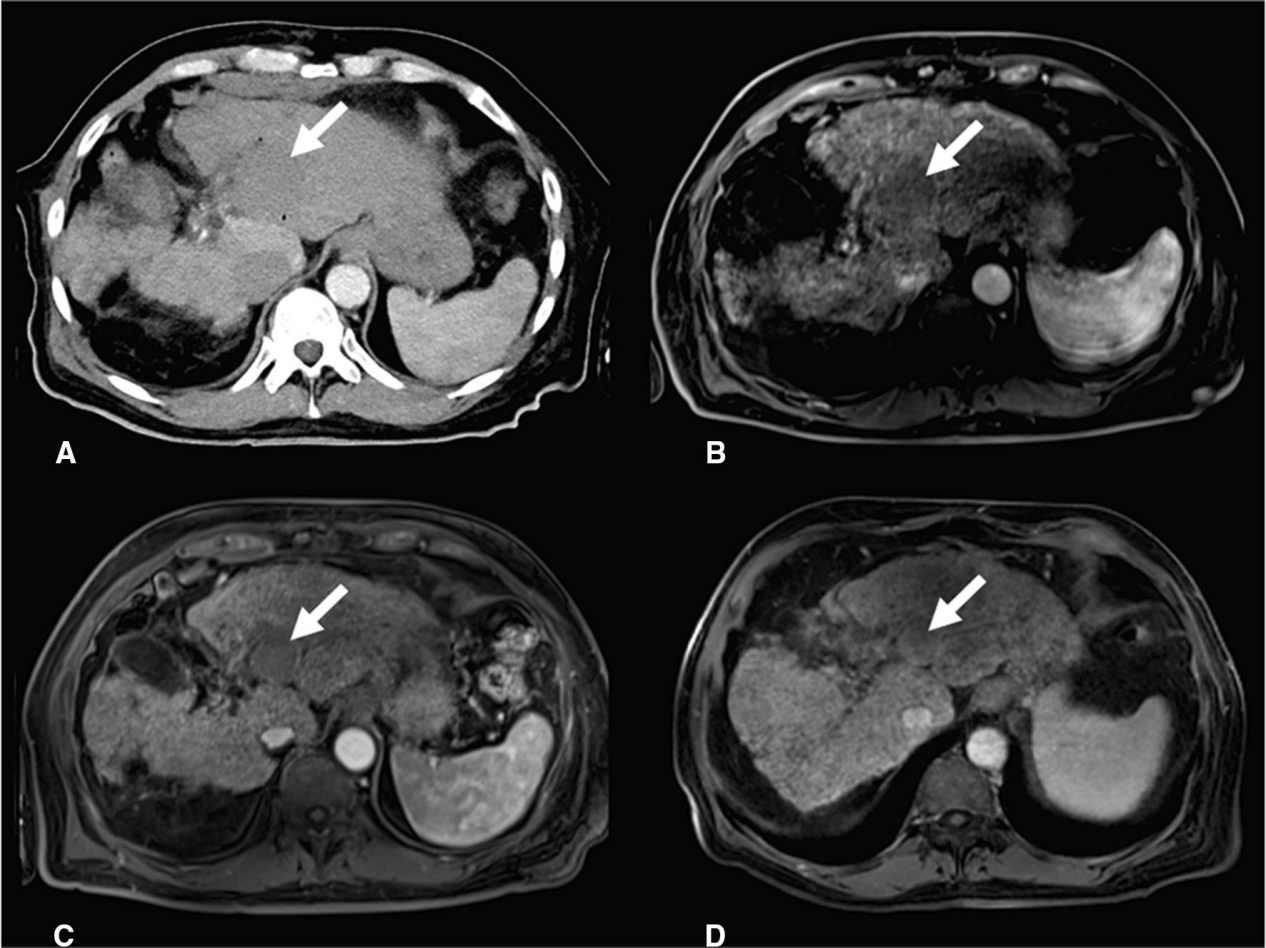
Follow-up imaging. **A** Contrast-enhanced CT scan in arterial phase at the end of intervention shows complete devascularization of the tumor (arrow). **B** Contrast-enhanced MRI in arterial phase, T1 Vibe 3d fs, 24 h after ablation confirms complete tumor devascularization (arrow). **C** and **D** Contrast-enhanced MRI in arterial phase, T1 Vibe 3d fs, 6 weeks (**C**) and 6 months (**D**) after ablation show complete response according to mRECIST and shrinking of the ablation defect to 3.1 × 2.8 × 2.8 cm without affection of the liver arteries and with almost complete restoration of the local cholestasis (arrow)

Contrast-enhanced MRI 6 weeks and 6 months after ECT showed complete response with no residual or recurrent HCC and without any signs of damage of the central bile ducts or affection of the liver artery (Fig. 3).

## Discussion

During open surgery, ECT has proven to be a potential method for treatment of HCC in 10 patients [10] as well as for treatment of colorectal liver metastases in 16 patients [9]. As of today, very little is known about percutaneous ECT of deep-seated tumors: only one case report on percutaneous ECT of a HCC lesion has been described [12]. In contrary to our case, Djocic et al. performed cone-beam computed tomography guided ECT on a small (18 mm), peripherally located HCC in a patient with multifocal HCC. That patient had already undergone TACE and MWA prior to ECT.

In our case ECT was performed using a primary stereotactic percutaneous approach for treatment of a large (4.7 × 4.5 × 3.5 cm) central unresectable HCC involving the major vessels and the main bile ducts. Multiantenna MWA was not suitable due to the central localization of the tumor with the associated risk of incomplete ablation due to heat sink effect from the hepatic hilar vessels and the risk of thermal damage to the central bile ducts [7]. IRE is more suitable to treat such central liver lesions but this technique is still limited to smaller lesions [7]. However, ECT allows the treatment of larger lesions, because the maximum distance between electrode pairs may be greater and still generate a sufficiently strong electric field (maximum distance of 3.0 cm per electrode pair for IGEA Cliniporator VITAE).

So far, ECT has never been combined with stereotactic navigation. A stereotactic percutaneous approach for ablation of liver lesions results in more precise positioning of the probes and the possibility to predict the exact ablation zones without extension of the procedure time [6]. By using a stereotactic percutaneous approach, we were able to position all probes for ECT precisely with an optimal distance between the electrode-tips ranging from 2.1 to 3.0 cm. Accordingly, the possibility to navigate the percutaneous transhepatic approach in accordance to the individual anatomy and to control it at any time using a stereotactic navigation system and computed tomography may be technically superior to ECT during open surgery or percutaneous techniques using fluoroscopy or cone-beam CT.

The short hospitalization in this case reflects the advantages of minimal-invasive percutaneous ablation therapy: median length of hospital stay after ECT during open surgery for liver metastases was 14 days [9].

## Conclusion

Stereotactic percutaneous ECT has the potential to be used as a curative treatment method for HCC with diameters of more than 4 cm, even in close proximity to critical structures like major blood vessels and central bile ducts.
